# Social Jetlag and Cardiometabolic Risk in Preadolescent Children

**DOI:** 10.3389/fcvm.2021.705169

**Published:** 2021-10-07

**Authors:** Nicholas Castro, Jake Diana, Jade Blackwell, James Faulkner, Sally Lark, Paula Skidmore, Michael Hamlin, Leigh Signal, Michelle A. Williams, Lee Stoner

**Affiliations:** ^1^School of Health and Applied Human Sciences, University of North Carolina, Wilmington, NC, United States; ^2^Department of Exercise and Sport Science, University of North Carolina at Chapel Hill, Chapel Hill, NC, United States; ^3^School of Sport, Health, and Community, University of Winchester, Winchester, United Kingdom; ^4^School of Sport, Exercise, and Nutrition, Massey University, Wellington, New Zealand; ^5^Department of Medicine, University of Otago, Dunedin, New Zealand; ^6^Department of Tourism, Sport and Society, Lincoln University, Christchurch, New Zealand; ^7^Sleep-Wake Research Centre, Massey University, Wellington, New Zealand; ^8^Department of Epidemiology, Harvard T.H. Chan School of Public Health, Boston, MA, United States

**Keywords:** circadian clock, social clock, activity behavior, sleep duration, vascular, social jetlag (SJL), metabolic

## Abstract

**Objective:** Childhood cardiometabolic disease risk (CMD) has been associated with short sleep duration. Its relationship with other aspects of sleep should also be considered, including social jetlag (SJL) which represents the difference between a person's social rhythms and circadian clock. This study investigated whether childhood CMD risk is associated with sleep duration, sleep disturbances, and SJL.

**Study Design:** The observational study included 332 children aged 8–10 years (48.5% female). The three independent variables were sleep duration, sleep disturbances, and SJL. SJL was calculated as the variation in hours between the midpoint of sleep during free (weekend) days and work/school days. Eleven cardiometabolic biomarkers were measured, including central blood pressure, lipids, glycated hemoglobin, arterial wave reflection, and glucose. Underlying CMD risk factors were identified using factor analysis.

**Results:** Four underlying CMD risk factors were identified using factor analysis: blood pressure, cholesterol, vascular health, and carbohydrate metabolism. Neither sleep disturbances nor sleep duration were significantly associated with any of the four CMD factors following adjustments to potential confounders. However, SJL was significantly linked to vascular health (*p* = 0.027) and cholesterol (*p* = 0.025).

**Conclusion:** These findings suggest that SJL may be a significant and measurable public health target for offsetting negative CMD trajectories in children. Further studies are required to determine biological plausibility.

## Introduction

Cardiometabolic disease (CMD) etiology is insidious, with the trajectory beginning during childhood or potentially pre-birth (1–4). Childhood is important because biological systems are highly malleable, responding to the interplay between genetic and environmental cues (3, 4). Environmental cues include exposure to the activity behaviors of sleep, sedentary time and physical activity (2–4). The importance of these activity behaviors during childhood has been emphasized through shifts in public health policy. For example, the recent Canadian 24-h Movement Guidelines advised on the amount of time children should engage in each activity behavior across the day in a digestible format (5). With respect to sleep, the guidelines recommend that children and youth (aged 5–13 years) should sleep for 9–11 h each night. However, while insufficient sleep length is related to increased CMD risk in children (6–9), other aspects of sleep, such as social jetlag (SJL), should be considered. Social jetlag is defined as the discrepancy between an individual's biological and social rhythms and, in children, is calculated as the total difference in the midpoints of sleep on weekdays vs. weekend days (10, 11).

Physiological processes, including glucose metabolism and blood pressure, display a circadian rhythm that is controlled by the internal circadian biological clock (12, 13). For children living in today's society, social obligations, particularly school schedules, can lead to SJL and disruption of circadian rhythms. Studies in children and early adolescence have reported associations between SJL and measures of body composition (14, 15). One of the studies found a statistically non-significant association between SJL and CMD risk (15). CMD risk was calculated as the linear sum of z scores for systolic blood pressure (SBP), high-density lipoprotein cholesterol (HDL), triglycerides (TG), waist circumference, and homeostatic model of insulin resistance (HOMA). This linear combination approach, which assumes equal weighting for each variable, is prone to multicollinearity and fails to recognize that some risk factors cluster (16). Alternatively, a data driven approach, such as factor analysis, can determine the appropriate weighting for each predictor, eliminate the problem of multicollinearity, and identify risk factor clusters (17).

This study investigated whether CMD risk in preadolescent children is associated with sleep duration, sleep disturbances, and SJL. Novel and conventional CMD risk stratification biomarkers were used in factor analysis to classify CMD risk factor clusters. We evaluated the hypothesis that SJL, independent of sleep duration and sleep disturbances, would be associated with CMD risk factor clusters.

## Methodology

This registered cross-sectional study (ACTRN12614000433606) meets the conditions of the New Zealand Health and Disability Ethics Committees (HDEC: 14/CEN/83), and accords with STROBE reporting guidelines (18).

### Participants

Between April 2015 and April 2016, children (aged 8–10 years) were recruited from schools within three regions (Canterbury, Otago, Wellington) across New Zealand. Schools were classified according to a decile system based on the predominant socioeconomic status (SES) of attending students. We categorized schools as low SES (Deciles 1–5) or high SES (Deciles 6–10), and randomly sampled from these strata. All children were eligible to participate, unless they had experienced orthopedic injury in the previous 3 months or had been prescribed cardiovascular medications. An information packet was provided to eligible children, and parental/guardian consent and child assent were obtained prior to data collection.

### Experimental Design

Data collection took place at the participating schools. Cardiometabolic measurements were made on a single day (Monday–Thursday) between the 9:00 and 12:00 o'clock hours. Children were asked to refrain from exercise for 24 h prior to measurement, be adequately hydrated, and have fasted for at least 3 h. The primary caregiver was asked to complete a questionnaire at home, which provided demographic and sleep habits data. Only children with complete sleep data and cardiometabolic health information were included in the analyses.

### Independent Variables

The three assessed sleep variables were sleep duration, sleep disturbances, and SJL. The participant's caregiver was asked to report the times their child typically rested and woke on both school days and free (weekend) days. The mean of sleep duration was calculated using a ratio of two weekend days to five weekdays. Social jetlag was estimated as the total discrepancy between the midpoints of sleep on weekend days vs. weekdays (10). Single items of customary school/weekday sleep display sufficient concurrent validity with actigraphy and diary records (19).

The Children's Sleep Habits Questionnaire (CSHQ) was utilized to estimate sleep disturbances, for which adequate test–retest reliability, discriminant validity, and internal consistency have been reported (20). The questionnaire consisted of 33 questions on a 7-point Likert-type scale ranging from Always (1) to Never (7) with higher scores indicative of greater sleep disturbances. The sum of all 33 scored CSHQ questions (with a potential range of 33–99), was used to calculate a Total Sleep Disturbance score. A Total Sleep Disturbance score >41 was applied to signify troubled sleep, as this cutoff has been revealed to precisely identify 80% of children with a clinically detected sleep disorder. Only the Total Sleep Disturbance score was used for this study (20).

### Dependent Variables

Eleven cardiometabolic variables were measured: central hemodynamics [augmentation index (AIx), central systolic blood pressure (cSBP), heart rate (HR)], peripheral hemodynamics [diastolic blood pressure (DBP) and SBP], cardiac blood markers [HDL, TG, total cholesterol (TC), low density lipoproteins (LDL)], metabolic blood markers [glycated hemoglobin (HbA1c) and fasting blood glucose (FBG)].

#### Peripheral and Central Hemodynamics

Pulse wave analysis (BP+, Uscom, Sydney, Australia) was applied to gauge all central hemodynamic variables. Oscillometric pressure waveforms were recorded by a single operator on the left upper arm following 20 min of undisturbed supine rest (21). Each measurement cycle was ~40 s, comprising a 10 s suprasystolic recording, followed by a brachial blood pressure recording. Utilizing a validated transfer function, an aortic pressure waveform was generated from which cSBP was estimated. Arterial wave reflection (AIx) was estimated from the suprasystolic waveform by means of the formula AIx = (P3 – P0)/(P1 – P0), where P0 denotes the pressure at the onset of the pulse, P1 the peak pressure of the incident wave, and P3 the peak pressure of the reflective wave. This index explains the relative height of the reflected pressure wave when compared to the incident waveform. The recordings with a quality signal were the only ones accepted (sign to noise ratio of >3dB). Two measurements were recorded, with a third if blood pressures differed by >5 mmHg or the AIx by >4%. Our group has reported acceptable between-day reliability for cSBP (ICC: 0.90), cSBP (ICC: 0.94) and AIx (ICC: 0.71) (22).

#### Cardiac and Metabolic Blood Markers

Capillary blood was collected to measure fasting HbA1c (A1CNow+, PTS Diagnostics) (23), and TG, FBG, TC, HDL and LDL (CardioChek PA, PTS Diagnostics) (23).

### Sample Size

Factor analysis was utilized to calculate the sample size, for which two dissimilar methods have been advocated based on: (i) the ratio of participants to variables, or (ii) a minimum total sample size (24). (ii) The recommended minimum participant to item ratio ranges from 5:1 to 10:1. For eleven variables, a minimum of 110 participants would be required using the 10:1 ratio. Minimum sample sizes, ranging from 50 to 400 have been recommended. Comfrey and Lee state that, “the adequacy of sample size might be evaluated very roughly on the following scale: 50–very poor; 100–poor; 200–fair; 300–good; 500–very good; 1000 or more–excellent” (24). The *n* = 332 for the current study meets Comfrey and Lee's “Good” criteria (24).

### Statistical Analysis

The Jamovi (v1.1.3.0) platform was used for statistical analysis. Participant demographic data were reported as mean and standard deviation. Regression outcomes are reported as beta (β) with 95% confidence intervals (95% CI). The significance level was set *a priori* for all statistical procedures at α = 0.05. The corresponding author (NC) had full access to the data in the study and was responsible for the integrity of the dataset. Anonymized data will be shared upon reasonable request.

CMD risk factors were identified using factor analysis (17). Factor analysis is a data-driven approach that can be used to identify how cardiometabolic variables cluster together. The assumption is that for a collection of observed variables there is a set of latent variables (factors) that can explain the interrelationships among those variables. This data-driven approach confers several advantages beyond simply summing *Z* scores: (i) accurate determination of predictor weighting; (ii) control for collinearity; and (iii) provision of additive information by the factors, beyond the individual components (17). The number of factors (i.e., latent variables) was determined by the minimum eigenvalue principle of greater than one for a factor analysis of the correlation matrix. The implication being that if an eigenvalue is greater than one the derived dimension captures less variability in the data than any single variable. Following orthogonal varimax rotation, the correlation between the derived factors and the underlying variables were used to interpret (label) each factor. We used a loading of >0.40 to interpret the factor pattern.

The associations between CMD risk and the three sleep measures were examined using linear regression analyses. To account for the clustered data (students within schools), we used Gaussian family generalized estimating equations with robust standard errors (25). Following the assessment of model assumptions, separate univariate analyses examined the association between each independent (average sleep duration, SJL, sleep disturbances) and each dependent variable (CMD risk factors), followed by a multivariable analysis. Two multivariable models were specified. Model 1 included the three sleep variables without further adjustments. Model 2 was additionally adjusted for school decile rating, ethnicity, age, and sex.

## Results

### Participants

**Figure 1:**
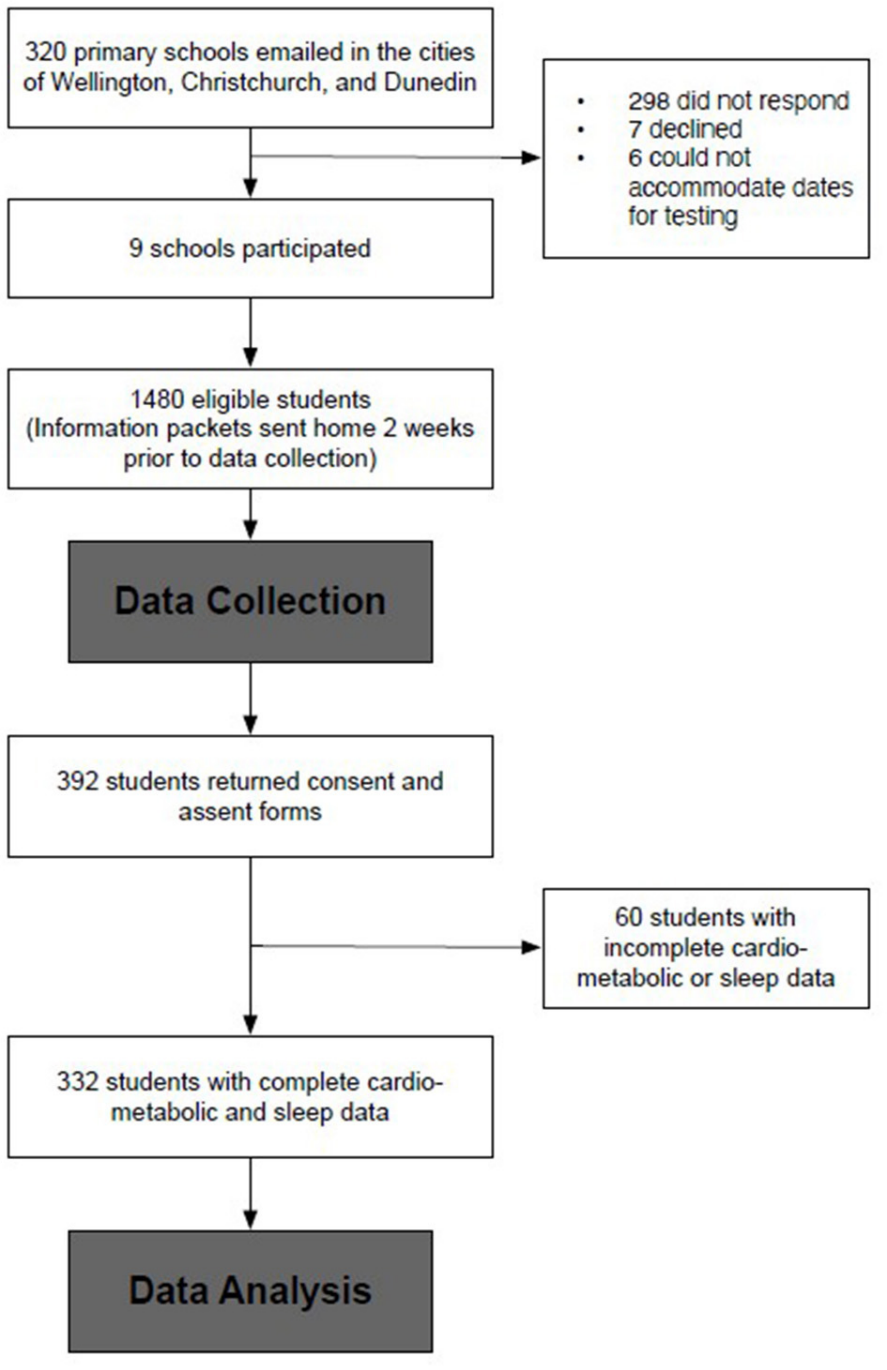
Student recruitment flow diagram.

Complete sleep and cardiometabolic data were available for *n* = 332 (Figure 1). These children were not discernibly different from the full 392 participants (9.6 ± 1.1 years, 48.5% female, 29.2% overweight, 51.5% low decile school). The demographic data are presented in Table 1. Most of the participants (92.8%, *n* = 308) slept for at least 9 h nightly, and the mean of the total sleep duration did not differ for the week compared to the weekend (10.1 vs. 10.1 h, *p* = 0.453). Less than half (39.2%) the students reported disturbed sleep. All but two of the participants that reported going to bed later on the weekend (mean: 42 min, 95% CI: 44, 50), and 97% (*n* = 330) also reported waking later on the weekend (42 min, 95% CI: 37, 46). Consequently, the average SJL was 44.4 ± 31.7 min, and 36.1% had >1-h SJL.

**Table 1:** Participant demographics stratified by sex (*n* = 332).

	**All**	**Male**	**Female**
**Categorical variables**	***n* (%)**	***n* (%)**	***n* (%)**
***n***	332 (100.0)	171 (51.5)	161 (48.5)
**Female**	161 (48.5)	0 (0.0)	161 (100.0)
**Ethnicity**			
New Zealand European	269 (81.0)	143 (83.6)	126 (78.3)
Māori	37 (11.1)	16 (9.4)	21 (13.0)
Pacific	22 (6.6)	10 (5.8)	12 (7.5)
Not recorded	4 (1.2)	2 (1.2)	2 (1.2)
**School year**			
4 (7–8 years old)	72 (21.7)	35 (20.5)	37 (23.0)
5 (8–9 years old)	93 (28.0)	51 (29.8)	42 (26.1)
6 (9–10 years old)	105 (31.6)	54 (31.6)	51 (31.7)
7 (10–11 years old)	62 (18.7)	31 (18.1)	31 (19.3)
**School decile**			
Low (≤ 5)	171 (51.5)	89 (52.0)	82 (50.9)
High (>5)	161 (48.5)	82 (48.0)	79 (49.1)
**Obesity status**			
Overweight-obese	235 (70.8)	121 (70.8)	114 (70.8)
Non-overweight	97 (29.2)	50 (29.2)	47 (29.2)
**Sleep**			
>9 h of sleep	308 (92.8)	159 (93.0)	149 (92.5)
>1 h of SJL	120 (36.1)	60 (35.1)	60 (37.3)
Sleep disorder	130 (39.2)	65 (38.0)	65 (38.0)
**Continuous variables**	**Mean (SD)**	**Mean (SD)**	**Mean (SD)**
**Sleep outcomes**			
WD sleep duration, hours	10.1 (0.89)	10.1 (0.89)	10.1 (0.91)
WE sleep duration, hours	10.1 (1.03)	10.1 (0.99)	10.1 (1.08)
Sleep duration, hours	10.1 (0.84)	10.1 (0.83)	10.1 (0.86)
Social jetlag, hours	0.74 (0.53)	0.68 (0.50)	0.80 (0.55)
**Cardiometabolic outcomes**			
Age, years	9.55 (1.13)	9.54 (1.06)	9.57 (1.20)
Systolic blood pressure, mmHg	101 (7.44)	101 (7.44)	101(8.18)
Diastolic blood pressure, mmHg	61.8 (6.24)	61.2(6.16)	62.4 (6.35)
Central systolic blood pressure, mmHg	93.5 (7.73)	93.2 (7.19)	93.7 (8.27)
Total cholesterol, mmol/L	3.57 (0.61)	3.5 (0.64)	3.64 (0.57)
Low-density lipoprotein cholesterol, mmol/L	1.85 (0.51)	1.78 (0.48)	1.91(0.53)
High-density lipoprotein cholesterol, mmol/L	1.47 (0.36)	1.51 (0.39)	1.43 (0.32)
Augmentation index, %	55.8 (15.2)	55.5 (15.0)	56.1 (15.5)
Heart rate, bpm	74.9 (11.5)	72 (10.2)	77.8 (12.3)
Fasting blood glucose, mmol/L	5.05 (0.39)	5.13 (0.39)	4.96 (0.38)
Triglycerides, mmol/L	1.47 (0.36)	0.839 (0.47)	0.892 (0.38)
Glycated hemoglobin, %	5.12 (0.31)	5.11 (0.31)	5.12 (0.31)

*WD, weekday; WE. weekend; n, sample size; mmHg, millimeters of mercury; mmol/L, millimoles per liter; %, percentage; SD, standard deviation, effect size (Cohen's d)*.

### Sleep and Cardiometabolic Disease Risk

The outcomes from factor analysis are reported in Table 2. The table displays the association of each variable with the four factors. The factors are labeled, in order of variance explained by the model: Blood Pressure, Cholesterol, Vascular Health and Carbohydrate Metabolism (CHO-MET). For example, Factor 1, which explains 23% of the cumulative variance, is labeled Blood Pressure because the variables which load most strongly onto the factor are the blood pressures.

**Table 2:** Factor analysis and eigenvalues (*n* = 332).

	**Factor 1**	**Factor 2**	**Factor 3**	**Factor 4**
	**Blood pressure**	**Cholesterol**	**Vascular**	**CHO-metabolism**
Systolic blood pressure	**0.90**	0.06	0.16	−0.01
Diastolic blood pressure	**0.89**	−0.01	0.04	−0.05
Central systolic blood pressure	**0.88**	−0.02	−0.08	0.04
Total Cholesterol	0.01	**0.92**	−0.01	0.07
Low density lipoproteins	0.02	**0.70**	0.03	0.03
High density lipoproteins	0.00	**0.55**	−0.14	**−0.45**
Augmentation index	0.18	−0.04	**−0.73**	0.02
Heart rate	0.27	0.10	**0.68**	0.02
Fasting blood glucose	0.04	−0.13	**0.51**	−0.05
Triglycerides	0.15	−0.10	−0.02	**0.73**
Glycated hemoglobin	−0.17	0.15	−0.10	**0.68**
Eigenvalue	2.5	1.7	1.3	1.2
% variance explained	23	16	12	11.0
Cumulative variance	23	39	51	62

*Bold numbers represent variables with a factor loading 0.4*.

Table 3 displays the univariate models. Sleep disturbances and sleep duration were not significantly associated with any CMD risk factor. Social jetlag was associated with cholesterol (β: −0.110, 95% CI: −0.416, −0.008) and vascular health (β: 0.162, 95% CI: 0.103, 0.501). Multivariable models 1 and 2 are shown in Table 3. Following adjustments for potential confounders (Model 2), SJL retained significant associations with cholesterol (β: −0.126, 95% CI: −0.448, −0.030) and vascular health (β: 0.125, 95% CI: 0.027, 0.438) risk factors.

**Table 3:** Linear associations between cardiometabolic risk factors and sleep measures (*n* = 332).

	**Univariate (*****n*** **=** **332)**					**Multivariate (Model 1) (*****n*** **=** **332)**					**Adjusted (Model 2) (*****n*** **=** **332)**				
	**β**	**LCI**	**UCI**	***P***	**ES**	**β**	**LCI**	**UCI**	***P***	**ES**	**β**	**LCI**	**UCI**	***P***	**ES**
**Factor 1. Blood pressure**															
Average sleep duration	0.002	−0.125	0.132	0.953	0.002	0.015	−0.113	0.147	0.793	0.012	0.026	−0.102	0.164	0.648	0.021
Sleep score	0.044	−0.011	0.025	0.425	0.263	0.040	−0.012	0.025	0.473	0.237	0.043	−0.011	0.025	0.442	0.254
Social jetlag	0.092	−0.029	0.377	0.093	0.049	0.091	−0.034	0.375	0.102	0.048	0.065	−0.086	0.332	0.251	0.501
**Factor 2. Cholesterol**															
Average sleep duration	−0.002	−0.132	0.127	0.969	−0.002	−0.013	−0.146	0.115	0.813	−0.010	0.019	−0.110	0.156	0.735	0.016
Sleep score	−0.034	−0.024	0.012	0.532	−0.205	−0.029	−0.023	0.013	0.604	−0.165	−0.022	−0.022	0.015	0.691	−0.130
Social jetlag	−0.110	−0.416	−0.008	0.041	−0.058	−0.111	−0.415	−0.005	0.045	−0.056	−0.126	−0.448	−0.030	0.025	−0.065
**Factor 3. Vascular health**															
Average sleep duration	−0.085	−0.226	0.027	0.122	−0.073	−0.069	−0.207	0.046	0.212	−0.058	−0.056	−0.197	0.065	0.321	−0.047
Sleep score	0.068	−0.007	0.029	0.215	0.415	0.048	−0.010	0.026	0.386	0.290	0.032	−0.013	0.032	0.557	0.196
Social jetlag	0.162	0.103	0.501	0.003	0.088	0.155	0.089	0.488	0.005	0.084	0.125	0.027	0.438	0.027	0.066
**Factor 4. CHO-MET**															
Average sleep duration	−0.058	−0.198	0.061	0.296	−0.048	−0.051	−0.192	0.069	0.357	−0.042	−0.026	−0.164	0.101	0.638	−0.022
Sleep score	0.017	−0.015	0.021	0.751	0.103	0.004	−0.018	0.019	0.948	0.022	−0.016	−0.021	0.015	0.776	−0.093
Social jetlag	0.097	−0.020	0.389	0.076	0.052	0.094	−0.027	0.384	0.089	0.049	0.031	−0.149	0.266	0.577	0.016

*Univariate: specify the sleep measures (sleep duration, sleep disturbances, and social jet lag) separately (separate mode); Model 1: unadjusted multivariable (sleep duration, sleep disturbances, and social jet lag); Model 2: Model 1 adjusted for age, sex, ethnicity, socioeconomic status.*

*n, sample size; β, beta; LCI, lower confidence interval; UCI, upper confidence interval; P, p-value; ES, effect size (Cohen's d)*.

## Discussion

The major finding of this study is that neither sleep disturbances nor sleep duration significantly correlated with any of the four CMD risk factors. However, SJL was significantly associated with cholesterol and vascular health factors. These findings suggest that SJL may be an important sleep characteristic with respect to CMD risk in children.

### Strengths and Limitations

The limitations and strengths of the current study are discussed here to provide context to the discussion that follows. First, we attempted to ensure the findings are generalizable to children across New Zealand by recruiting participants from three regions across the country. However, the findings may not generalize to populations outside of New Zealand. Second, this was a cross-sectional study and association is not causality. Longitudinal and intervention analyses are warranted to validate the results of the current study. Third, parent-report questionnaires were used to collect the sleep data rather than objective measures. While concurrent validity has been reported for the single item sleep surveys compared to actigraphy and diary data (19), future studies with objective assessments are warranted. Last, while our CMD risk model did include both FBG (short term glycemic control) and HbA1c (chronic glycemic control), insulin was not measured. There is some evidence that, at least in obese children, hyperinsulinemia precedes impaired fasting glucose control (13, 26). Future CMD risk models may be improved through the addition of insulin. The key strengths included the use of a data-driven approach for characterizing CMD risk, the co-assessment of three sleep variables thought to be important to a child's health, and our relatively large and representative cohort of New Zealand-based preadolescents.

### Comparison to the Literature

A number of studies in adults have reported positive (i.e., negative) associations between CMD risk and SJL (13, 26, 27). Though only one known study has examined the association between CMD risk and SJL in children (15). Cespedes Feliciano et al. (15) reported a non-significant association between CMD risk *Z* scores and SJL for both girls (β = 0.07, 95% CI: −0.02 to 0.15) and boys (β = 0.00, 95% CI: −0.08 to 0.08). One potential explanation for the conflicting findings between the Cespedes Feliciano et al. study (15) and current study is the difference in sample characteristics, including the recruitment of older children (12–17 year vs. 8–10 years, respectively). However, considering CMD risk has been associated with SJL in various adult populations this explanation is unlikely. A more likely explanation pertains to methodological differences. For the current study, we measured a comprehensive and diverse mix of eleven cardiometabolic parameters and used factor analysis to identify risk factor clusters (17). Using this approach we found that SJL was associated with the factors we labeled “cholesterol” and “vascular health,” but not “blood pressure” and “CHO-metabolism.” Cespedes Feliciano et al. (15) divided their sample of 479 adolescents by sex (breakdown not provided) and characterized CMD risk using composite z scores for waist circumference, SBP, HDL, TG, and HOMA. This linear combination method would lack the sensitivity of the data-driven approach used in the current study as it assumes equal weighting for each variable, is prone to multicollinearity, and fails to recognize that risk factors cluster (16).

SJL, but not sleep duration or sleep disturbances, was associated with CMD risk. This finding is particularly surprising considering that both sleep-related problems and short sleep duration have previously been associated with CMD risk (6–9). One potential explanation is the lack of co-consideration of SJL in previous studies. However, a more likely explanation is statistical in nature, particularly with respect to sleep duration. In the current study, 93% of children reported having slept for the recommended >9 h each night. While these data were self-reported, it remains highly likely that the number of short sleepers in the current study was low. The restricted range in the data likely limited the capacity to detect an association between sleep duration and CMD risk (28). Therefore, future pediatric sleep research should consider utilizing more objective measures of sleep in children (e.g., actigraphy).

We found that SJL correlated with vascular health and cholesterol factors, but not the blood pressure or CHO-metabolism factors. This finding is perhaps somewhat surprising when considering that SJL may lead to disruption of circadian rhythms in glucose metabolism and blood pressure, and have negative consequences for the regulation of these processes (12, 13). However, it should be acknowledged that while the vascular health factor was primarily driven by AIx, a measure of arterial wave reflection, FBG did load on to this factor. It is plausible that SJL disrupts metabolic pathways, and that these disruptions impair vascular function and hemodynamic parameters, including increased arterial wave reflection. Further research is warranted to determine biological plausibility for the relationship between SJL and CMD risk in children.

### Implications

There has been a recent shift in public health policy to acknowledge childhood activity behaviors in the context of the entire 24 h day (5). With respect to sleep, it is recommended that children and youth sleep for 9–11 h each night. Even though short sleep duration is linked to increased CMD risk in children (6–9), there are other aspects of sleep that should also be considered in the context of 24 h activity behavior. In particular, findings from the current study suggest that SJL contributes to CMD in children. The current findings are strengthened by the fact that 93% of the children in the current study reported having slept the recommended minimum 9 h of sleep (5). Despite most children achieving an adequate duration of sleep, SJL remained associated with CMD risk. Considering that SJL is arguably a readily modifiable target, i.e., ensuring children maintain consistent and chronotype-driven sleep–wake cycles, further research is warranted to ascertain causality and to determine whether SJL-based interventions improve cardiometabolic outcomes. Another area that warrants future attention is the exploration of sex-specific effects. In the current study, we did not stratify our data by sex as the smaller sample would have been insufficient for the factor analysis. Our group and others have acknowledged sex to moderate the relationship between SJL and health outcomes, such as body composition (14, 15) and cardiorespiratory fitness (29). Hence, the causal relationship between SJL and CMD risk may differ by sex justifying the need for tailored sex-specific interventions.

## Conclusions

This study examined the independent associations between sleep disturbances, sleep duration, and SJL with CMD risk in pre-adolescent children. We found that SJL, but not sleep duration or sleep disturbances, was associated with CMD risk. These results suggest that SJL may be a significant and quantifiable public health target for offsetting negative CMD trajectories in children.

## Data Availability Statement

The original contributions presented in the study are included in the article/supplementary material, further inquiries can be directed to the corresponding author/s.

## Ethics Statement

The studies involving human participants were reviewed and approved by New Zealand Health and Disability Ethics Committee (HDEC: 14/CEN/83). Written informed consent to participate in this study was provided by the participants' legal guardian/next of kin.

## Author Contributions

NC, JF, SL, MW, and LSt conceived of the present idea and contributed to the design and implementation of the research. NC planned and performed the experiments. NC and LSt developed the theory and performed the computations. MH, LSt, and PS performed the numerical calculations for the suggested experiment and verified the analytical methods. NC, JD, and JB took the lead in writing the manuscript. All authors discussed the results and provided critical feedback to the writing of the manuscript.

## Funding

This study was supported by New Zealand International Doctoral Research Scholarship Recipient (2014–2017) and Massey University Doctoral Research Scholarship Recipient (2014–2017).

## Conflict of Interest

The authors declare that the research was conducted in the absence of any commercial or financial relationships that could be construed as a potential conflict of interest.

## Publisher's Note

All claims expressed in this article are solely those of the authors and do not necessarily represent those of their affiliated organizations, or those of the publisher, the editors and the reviewers. Any product that may be evaluated in this article, or claim that may be made by its manufacturer, is not guaranteed or endorsed by the publisher.

## Abbreviations

AIx: augmentation index
β: beta
cSBP: central systolic blood pressure
CSHQ: Children's Sleep Habits Questionnaire
DBP: diastolic blood pressure
FBG: fasting blood glucose
HbA1c: glycated hemoglobin
HDL: high density lipoproteins
HR: heart rate
LDL: low density lipoproteins
mmHg, mmol/L: millimoles per liter
%: percentage
mmHg: millimeters of mercury
*n*: sample size
SBP: systolic blood pressure
SD: standard deviation
SES: socioeconomic scale
SJL: Social jetlag
TC: total cholesterol
STROBE: Strengthening the Reporting of Observational Studies in Epidemiology
TG: triglycerides
WD: weekday
WE: weekend.

## References

[1] Agarwal P Morriseau TS Kereliuk SM Doucette CA Wicklow BA Dolinsky VW. Maternal obesity, diabetes during pregnancy and epigenetic mechanisms that influence the developmental origins of cardiometabolic disease in the offspring. Crit Rev Clin Lab Sci. (2018) 55:71–101. 10.1080/10408363.2017.1422109 29308692

[2] Stoner L Kucharska-Newton A Meyer ML. Cardiometabolic health and carotid-femoral pulse wave velocity in children: a systematic review and meta-regression. J Pediatr. (2020) 218:98–105.e3. 10.1016/j.jpeds.2019.10.065 31810627 PMC7260444

[3] Stoner L Matheson A Hamlin M Skidmore P. Environmental determinants of childhood obesity: a specific focus on Maori and Pasifika in New Zealand. Perspect Public Health. (2016) 136:18–20. 10.1177/1757913915616734 26702112

[4] Wu BW Skidmore PM Orta OR . Genotype vs. phenotype and the rise of non-communicable diseases: the importance of lifestyle behaviors during childhood. Cureus. (2016) 8:e458. 10.7759/cureus.458 26918226 PMC4752369

[5] Tremblay MS Carson V Chaput J-P. Introduction to the Canadian 24-hour movement guidelines for children and youth: an integration of physical activity, sedentary behaviour, and sleep. Appl Physiol Nutr Metab. (2016) 41:iii–iv. 10.1139/apnm-2016-0203 27306430

[6] Stoner L Higgins S Black K Boggess K Meyer ML Chou A. Short sleep duration is associated with central arterial stiffness in children independent of other lifestyle behaviors. J Sci Sport Exerc. (2020) 2:236–45. 10.1007/s42978-020-00062-5

[7] Lee JA Park HS. Relation between sleep duration, overweight, and metabolic syndrome inKorean adolescents. Nutr Metab Cardiovasc Dis. (2014) 24:65–71. 10.1016/j.numecd.2013.06.004 24188647

[8] Pulido-Arjona L Correa-Bautista JE Agostinis-Sobrinho C Mota J Santos R Correa-Rodríguez M. Role of sleep duration and sleep-related problems in the metabolic syndrome among children and adolescents. Ital J Pediatr. (2018) 44:1–10. 10.1186/s13052-018-0451-7 29334985 PMC5769404

[9] Seo SH Shim YS. Association of sleep duration with obesity and cardiometabolic risk factors in children and adolescents: a population-based study. Sci Rep. (2019) 9:1–10. 10.1038/s41598-019-45951-0 31263172 PMC6603036

[10] Roenneberg T Allebrandt K V., Merrow M, Vetter C. Social jetlag and obesity. Curr Biol. (2012) 22:939–43. 10.1016/j.cub.2012.03.038 22578422

[11] Stoner L Beets MW Brazendale K Moore JB Weaver RG. Social jetlag is associated with adiposity in children. Glob Pediatr Heal. (2018) 5:2333794X18816921. 10.1177/2333794X18816921 30547059 PMC6287324

[12] Arble DM Bass J Behn CD. Impact of sleep and circadian disruption on energy balance and diabetes: a summary of workshop discussions. Sleep. (2015) 38:1849–60. 10.5665/sleep.5226 26564131 PMC4667373

[13] Wong PM Hasler BP Kamarck TW Muldoon MF Manuck SB. Social jetlag, chronotype, and cardiometabolic risk. J Clin Endocrinol Metab. (2015) 100:4612–20. 10.1210/jc.2015-2923 26580236 PMC4667156

[14] Stoner L Castro N Signal L. Sleep and adiposity in preadolescent children: the importance of social jetlag. Child Obes. (2018) 14:158–64. 10.1089/chi.2017.0272 29298086

[15] Cespedes Feliciano EM Rifas-Shiman SL Quante M Redline S Oken E Taveras EM. Chronotype, social jet lag, and cardiometabolic risk factors in early adolescence. JAMA Pediatr. (2019) 94612:1–9. 10.1001/jamapediatrics.2019.3089 31524936 PMC6749538

[16] Reaven GM. Banting lecture 1988. Role of insulin resistance in human disease. Diabetes. (1988) 37:1595–607. 10.2337/diabetes.37.12.1595 3056758

[17] Stoner L Weatherall M Skidmore P Castro N Lark S Faulkner J. Cardiometabolic risk variables in preadolescent children: a factor analysis. J Am Heart Assoc. (2017) 6:e007071. 10.1161/JAHA.117.007071 29021277 PMC5721889

[18] von Elm E Altman DG Egger M. The strengthening the reporting of observational studies in epidemiology (STROBE) statement: guidelines for reporting observational studies. Lancet. (2007) 370:1453–7. 10.1016/S0140-6736(07)61602-X 18064739

[19] Wolfson AR Carskadon MA Acebo C Seifer R Fallone G Labyak SE. Evidence for the validity of a sleep habits survey for adolescents. Sleep. (2003) 26:213–6. 10.1093/sleep/26.2.213 12683482

[20] Owens JA Spirito A McGuinn M Nobile C. Sleep habits and sleep disturbance in elementary school-aged children. J Dev Behav Pediatr. (2000) 21:27–36. 10.1097/00004703-200002000-00005 10706346

[21] Stoner L Lambrick DM Faulkner J Young J. Guidelines for the use of pulse wave analysis in adults and children. J Atheroscler Thromb. (2013) 20:404–6. 10.5551/jat.16295 23358124

[22] Stoner L Lambrick DM Westrupp N Young J Faulkner J. Validation of oscillometric pulse wave analysis measurements in children. Am J Hypertens. (2014) 27:865–72. 10.1093/ajh/hpt243 24390294

[23] Parikh P Mochari H Mosca L. Clinical utility of a fingerstick technology to identify individuals with abnormal blood lipids and high-sensitivity C-reactive protein levels. Am J Health Promot. (2009) 23:279–82. 10.4278/ajhp.071221140 19288850 PMC2750040

[24] Osborne JW Costello AB. Sample size and subject to item ratio in principal components analysis. Pract Assessment Res Eval. (2004) 9:1–9. Available online at: 10.7275/ktzq-jq66

[25] Goldstein H. Multilevel Statistical Models. John Wiley & Sons, Ltd. (2010). Chichester, UK.

[26] Islam Z Akter S Kochi T Hu H Eguchi M Yamaguchi M. Association of social jetlag with metabolic syndrome among Japanese working population: the Furukawa Nutrition and Health Study. Sleep Med. (2018) 51:53–8. 10.1016/j.sleep.2018.07.003 30099352

[27] Zuraikat FM Makarem N Redline S Aggarwal B Jelic S St-Onge MP. Sleep regularity and cardiometabolic heath: is variability in sleep patterns a risk factor for excess adiposity and glycemic dysregulation? Curr Diab Rep. (2020) 2:38. 10.1007/s11892-020-01324-w 32700156 PMC7584347

[28] Bland JM Altman DG. Correlation in restricted ranges of data. BMJ. (2011) 342:d556. 10.1136/bmj.d556 21398359

[29] Higgins S Stoner L Lubransky A. Social jetlag is associated with cardiorespiratory fitness in male but not female adolescents. Sleep Med. (2020) 75:163–70. 10.1016/j.sleep.2020.07.030 32858356

